# Supplementary material for: Imaging the impact of rotifer consumption on bacterial behaviors in the zebrafish gut
Source: PLoS One. 2026 Jun 2;21(6):e0349516. doi: 10.1371/journal.pone.0349516 (PMC13229379; doi:10.1371/journal.pone.0349516)
Supplement: S1 File — Supplemental Movie Captions, Supplemental Figures and Captions, Tables of strains, plasmids, and primers used for fluorescent T6SS activity reporter construction. (PDF) [file pone.0349516.s001.pdf]

Supplemental Material

Imaging the impact of rotifer consumption on bacterial behaviors in the zebrafish gut

Susana Márquez Rosales[1], Piyush Amitabh[1], Emily M. Olmstead[1], Emily P. R. Avey[1], Elena S. Wall[2], Lizett Ortiz de Ora[3], Travis J. Wiles[3], and Raghuveer Parthasarathy[1,*]

1 Department of Physics, Institute of Molecular Biology, and Materials Science Institute, University of Oregon, Eugene, Oregon, 97403, USA
2 Institute of Molecular Biology, University of Oregon, Eugene, Oregon, 97403, USA
3 Department of Molecular Biology and Biochemistry, University of California, Irvine, California, 92697, USA
*Corresponding author: raghu@uoregon.edu

Contents

- Description of Supplemental Data
- Supplemental Movie Captions
- Supplemental Figures and Captions
- Tables of strains, plasmids, and primers used for fluorescent T6SS activity reporter construction

Description of Supplemental Data

The following are contained in a compressed (ZIP) file, supplementary_data.zip:

Plotted Datapoints. CSV files of all plotted datapoints, organized by figure.

Supplemental Movie Captions

Supplemental Movie 1. Animated z-stack of light sheet fluorescence microscopy images of unfed larval anterior gut and surrounding regions, shown in Figure 1B. Cyan: green autofluorescence, magenta: red autofluorescence as explained in the main text.

Supplemental Movie 2. Animated z-stack of light sheet fluorescence microscopy images of fed larval anterior gut and rotifers, shown in Figure 1C. Cyan: green autofluorescence, magenta: red autofluorescence as explained in the main text.

Supplemental Movie 3. A series of maximum intensity projections of 3D images taken from time lapse, every 15 minutes, imaging rotifers transit within a fed larval zebrafish gut (Figure 1E). Composite overlays: cyan shows green autofluorescence and magenta shows red autofluorescence, shown in Figure 1E.

Supplemental Movie 4. Animated z-stack of light sheet fluorescence microscopy images of unfed zebrafish intestines (6 dpf) showing GFP-labeled Enterobacter (EN) (cyan) and red autofluorescence from the gut (magenta), shown in Figure 2A.

Supplemental Movie 5. Animated z-stack of light sheet fluorescence microscopy images of fed zebrafish intestines (6 dpf) showing GFP-labeled *Enterobacter* (cyan) and red autofluorescence from the rotifers (magenta), shown in Figure 2B.

Supplemental Movie 6. Light sheet fluorescence microscopy movie of motile *Enterobacter* within a fed larva. Each frame is from the same optical plane, which spans the midgut region (Fig. 2B).

Supplemental Movie 7. A series of maximum intensity projections of 3D images taken from time lapse, every 15 minutes, imaging GFP-labeled *Enterobacter* (cyan) and rotifers transit (autofluorescence in magenta) within a fed larval zebrafish gut (Figure 2I). After rotifers reach the vent, *Enterobacter* forms aggregates again.

Supplemental Movie 8. Animated z-stack of light sheet fluorescence microscopy images of unfed zebrafish intestines (6 dpf) showing GFP-labeled *Vibrio* (cyan) and red autofluorescence from the gut (magenta), shown in Figure 3A.

Supplemental Movie 9. Animated z-stack of light sheet fluorescence microscopy images of fed zebrafish intestines (6 dpf) showing GFP-labeled *Vibrio* (cyan) and red autofluorescence from the rotifers (magenta), shown in Figure 3B.

Supplemental Movie 10. Light sheet fluorescence microscopy movie of motile *Vibrio* in an unfed larva. Each frame is from the same optical plane, which spans the midgut region (Fig. 3A).

Supplemental Movie 11. Light sheet fluorescence microscopy movie of motile *Vibrio* within a fed larva. Each frame is from the same optical plane, which spans the midgut region (Fig. 3B). The most bright non-motile regions correspond to ingested rotifers.

Supplemental Figures

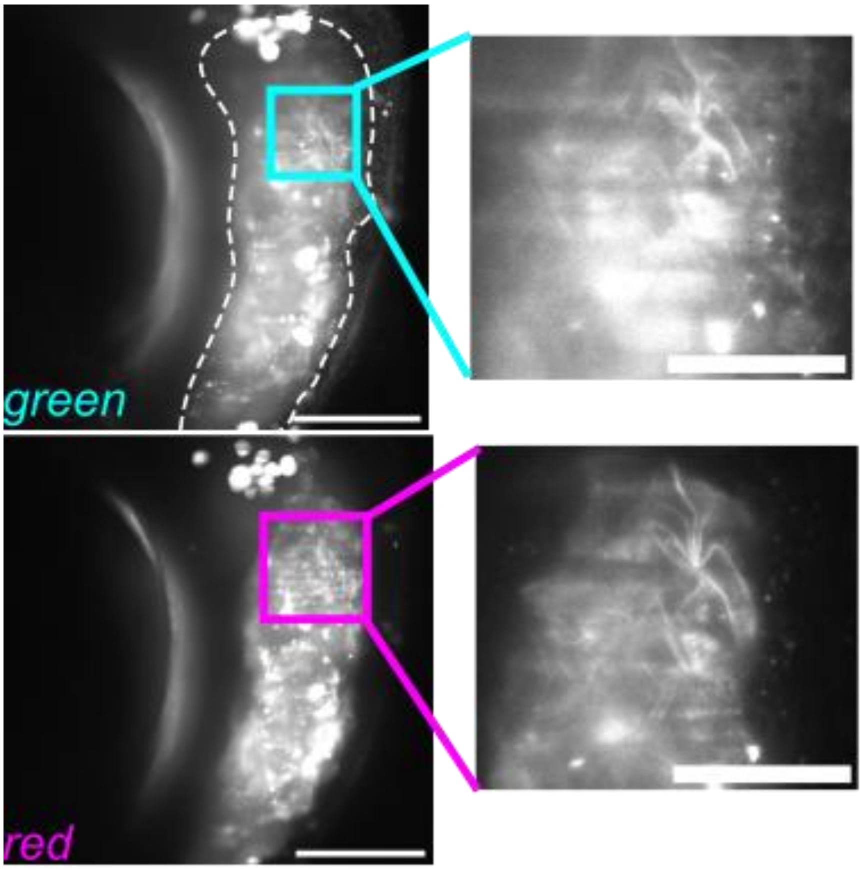

Supplemental Figure S1. Anterior gut of fed zebrafish autofluorescence imaged with light-sheet fluorescence microscopy (LSFM) using blue/green excitation/emission wavelengths (excitation 488 nm, emission 510-550 nm, denoted 'green') and autofluorescence using green/red excitation/emission wavelengths (excitation 561 nm, emission 590-650 nm, denoted 'red').

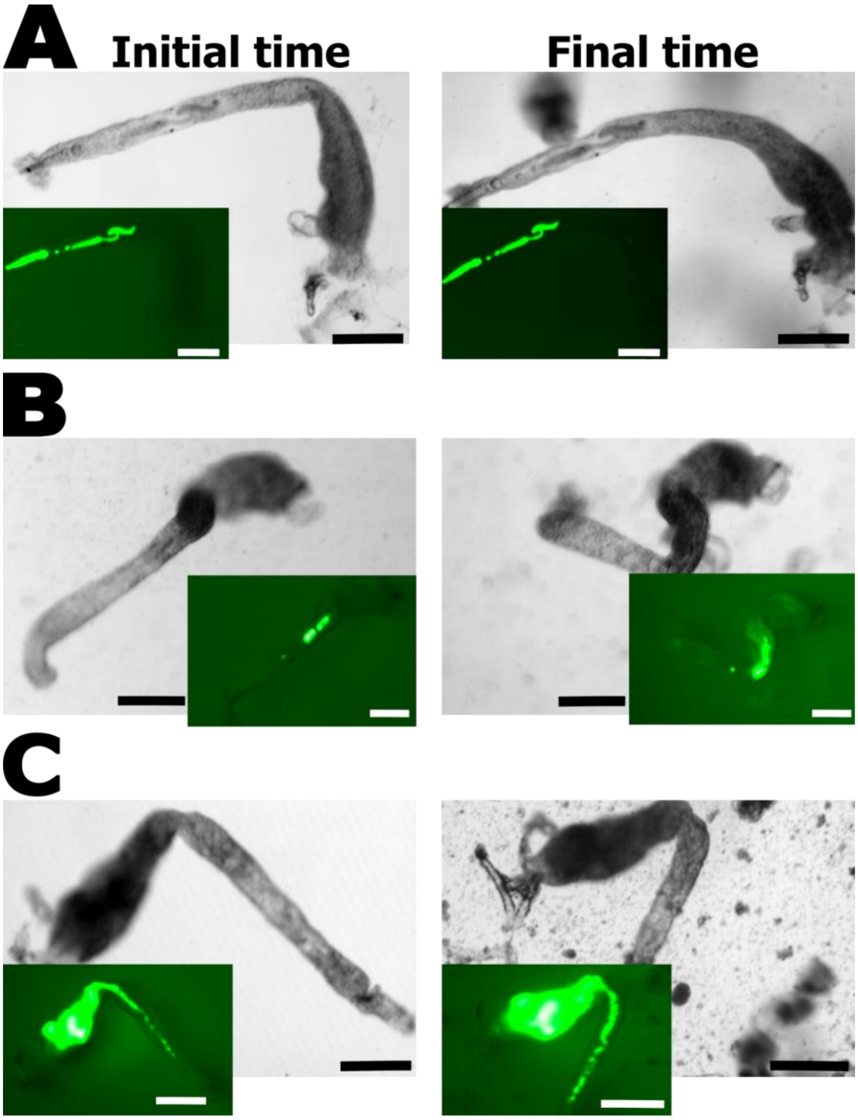

Supplemental Figure S2. Dissected guts of zebrafish larvae containing fluorescent EN aggregates. Panels A–C correspond to different larvae. Left: Initial time, right: final time. The gray picture shows the bright field image and the green images show the *Enterobacter* GFP. Scale bar: 200 μm.

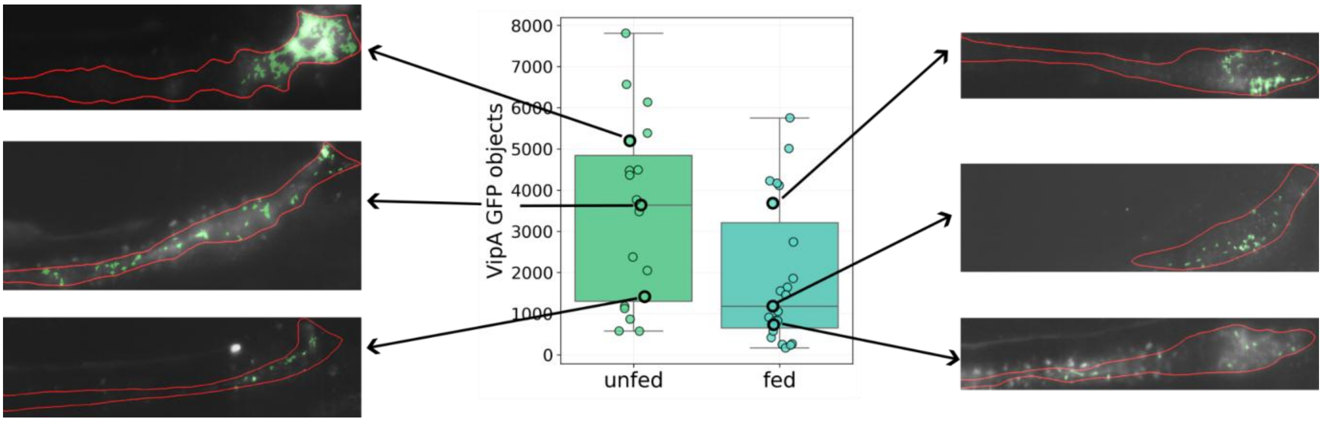

Supplemental Figure S3 Examples of segmented VipA-GFP objects in unfed and fed larvae (see Figure 4E). The red line outlines the gut mask; green regions are segmented objects.

Tables of strains, plasmids, and primers used for fluorescent T6SS activity reporter construction

Strains

Strain	Description/ Relevant Details	Reference/Source
DH5α	general cloning strain	NEB
SM10(λpir)	pir^+ mating strain; Kan^R	REF
Vibrio ZWU0020	*Vibrio cholerae* strain isolated healthy zebrafish gut	

Plasmids.

Plasmid	Description/ Relevant Details	Reference/Source
pAX2	allelic exchange vector with $GFP^{mut3.1}$ merodiploid tracker, temperature-sensitive replicon $ori_{101}/repA101^{ts}$, and TetR-controlled kill switch; Amp^R, $Gent^R$, Clm^R, 30°C	Wiles et al, 2018
pTW469	pAX2 with an allelic exchange cassette containing the *vipA-sfGFP* fusion; Amp^R, $Gent^R$, Clm^R, 30°C (Strain # TW469)	This study (or Cathy's?)
pmScarlet_C1	pC1 vector with monomeric (m)Scarlet template; obtained as a gift from Dorus Gadella (Addgene plasmid # 85042; http://n2t.net/addgene:85042; RRID:Addgene_85042)	REF
pXS-sfGFP	Vector with a modular sfGFP expression scaffold; Amp^R	Wiles et al, 2018
pXS-mScarlet	pXS vector with constitutive mScarlet gene (Strain # pTTW3)	
pTn7xTS	Tn*7* tagging vector with temperature-sensitive replicon $ori_{101}/repA101^{ts}$; Amp^R, $Gent^R$, 30°C	Wiles et al, 2018
pTn7xTS-mScarlet	Tn*7* tagging vector pTn7xTS temperature-sensitive replicon $ori_{101}/repA101^{ts}$; Amp^R, $Gent^R$, 30°C (Strain # TTW8)	This study
pTNS2	pTNS2; Tn*7* helper plasmid carrying Tn*7* transposase genes; Amp^R	REF

Primer sequences.

Name	Sequence (5'–3')

seq.pUC18R6KT-Tn7T-T1-REV (WP17)	cttaaacgcctggggtaatg
hokB.Ptac_SOE.FOR (WP24)	tgagcggataacaatttcacacaggagaaaggctatgaagcac
SFGFP.REV (WP126)	tcacttgtacagctcgtccatg
seq.sfGFP.5p.REV (WP140)	ctgaacttgtggccgtttac
5p.HR.SmaI.vipA.FOR (WP245)	cccgggtattagatcatcgcaaaca
5p.HR.vipA.3A3G.sfGFP.SOE.REV (WP246)	agctcctcgcccttgctcataccaccgcccgcagctgccgcttgtggctcttcttgac
3A3G.sfGFP.SOE.vipA.FOR (WP247)	gtcaagaagagccacaagcggcagctgcgggcggtggtatgagcaagggcgaggagct
sfGFP.SOE.vipA.REV (WP248)	gtgattaattgattaataacgtttgctcacttgtacagctcgtccatg
3p.HR.sfGFP.vipA.SOE.FOR (WP249)	catggacgagctgtacaagtgagcaaacgttattaatcaattaatcac
3p.HR.SmaI.vipA.REV (WP250)	cccgggtggattttcgattggatcgt
seq.vipA.FOR (WP251)	ccgagcttcctgttgaactc
seq.vipA.3p.HR.REV (WP252)	tgcagcagcttcatatctgg
mScarlet.IVA.pXS.F	tttgtttaactttaagaaggagatatacatatggtgagcaagggcgag
mScarlet.IVA.pXS.R	tttgtagagctcatccatgccatgtgtgcggccgcttacttgtacagctcgtccatgcc
Tn7R.PCRver.FOR (WP11)	cacgcccctctttaatacga
Tn7.insert.ZWU0020.2.FOR (WP12)	agggtaccgatgttgaccag
